# A Higher Burden of Abnormal Antioxidant-, Nutritional-, and Inflammation-Related Biomarkers Is Associated with Shoulder-Hand Syndrome, Hand Edema, and Short-Term Upper-Limb Outcomes During Stroke Rehabilitation

**DOI:** 10.3390/antiox15091173

**Published:** 2026-09-16

**Authors:** Ki-Hyeok Ku, Eo Jin Park

**Affiliations:** 1 Department of Orthopedic Surgery, Kyung Hee University College of Medicine, Kyung Hee University Hospital at Gangdong, Seoul 05278, Republic of Korea; 2 Department of Rehabilitation Medicine, Kyung Hee University College of Medicine, Kyung Hee University Hospital at Gangdong, 892 Dongnam-ro, Gangdong-gu, Seoul 05278, Republic of Korea

**Keywords:** antioxidant-, nutritional-, and inflammation-related biomarker burden, stroke, shoulder-hand syndrome, hand edema, zinc, rehabilitation, Fugl-Meyer upper extremity, C-reactive protein, neutrophil-to-lymphocyte ratio

## Abstract

Post-stroke shoulder-hand syndrome and hand edema can complicate inpatient rehabilitation and may reflect interactions between antioxidant, nutritional, inflammatory, and neuro-musculoskeletal factors. This single-center retrospective cohort study included 453 adults admitted for inpatient rehabilitation within 60 days after a first-ever unilateral ischemic or hemorrhagic stroke between 1 January 2018 and 31 December 2025. The primary exposure was the number of abnormal antioxidant-, nutritional-, and inflammation-related biomarkers, defined as serum zinc <70 µg/dL, albumin <3.5 g/dL, C-reactive protein ≥1.0 mg/dL, and neutrophil-to-lymphocyte ratio ≥3.0. The primary outcome was shoulder-hand syndrome or clinically significant hand edema during inpatient rehabilitation. In multivariable logistic regression with robust standard errors, each 1-unit increase in the number of abnormal biomarkers was associated with higher odds of the primary composite outcome (odds ratio, 1.43; 95% confidence interval, 1.15–1.77; *p* = 0.001). Directionally consistent associations were observed for shoulder-hand syndrome, clinically significant hand edema, Fugl–Meyer upper-extremity score at discharge, and poor upper-limb function at discharge. In a temporal sensitivity analysis excluding the 28 patients with the primary outcome already documented at PM&R admission (*N* = 425), biomarker profiling preceded the documented outcome in all 98 subsequent event cases, and the association remained (odds ratio, 1.42; 95% confidence interval, 1.12–1.79; *p* = 0.004). These findings indicate that a higher burden of abnormal antioxidant-, nutritional-, and inflammation-related biomarkers was associated with a clinical profile characterized by upper-limb complications and less favorable short-term motor function; the burden measure is not validated for prediction.

## 1. Introduction

Stroke rehabilitation aims to reduce disability while preventing complications that interfere with recovery of the affected upper limb [1,2,3]. Shoulder-hand syndrome and clinically significant hand edema are common clinical concerns during inpatient rehabilitation because they may aggravate pain, restrict therapeutic participation, limit range-of-motion training, and complicate the assessment of motor recovery [4,5,6]. These problems are often interpreted through neuro-musculoskeletal mechanisms such as paresis, shoulder subluxation, altered autonomic regulation, immobility, and spasticity [7,8]. However, clinical heterogeneity suggests that some patients present with disproportionate swelling or pain despite similar motor severity, raising the possibility that systemic biological factors may also contribute to post-stroke upper-limb complications [7,9].

Oxidative stress, nutritional depletion, and inflammation are biologically plausible contributors to tissue edema, nociceptive sensitization, endothelial dysfunction, and post-stroke recovery [10,11,12]. Serum zinc (Zn) is an antioxidant-related trace element that plays a role in immune regulation, redox homeostasis, and tissue repair [13,14,15]. Albumin partly reflects nutritional reserve and antioxidant capacity, whereas C-reactive protein (CRP) and the neutrophil-to-lymphocyte ratio provide accessible measures of systemic inflammatory activation [16,17,18,19]. In patients undergoing stroke rehabilitation, these biomarkers are available in many clinical settings and may capture different and partly overlapping dimensions of antioxidant and inflammatory burdens [20,21,22]. Rather than relying on a single laboratory marker, a combined count of abnormal biomarkers may provide a pragmatic summary of the multidomain laboratory profile around rehabilitation admission. These biomarkers were therefore treated as related but biologically distinct indicators rather than as components of a single unified biological phenotype.

Previous studies have addressed individual biomarkers in relation to stroke severity, neurological prognosis, infection, nutritional status, and functional outcomes [20,21,23,24]. Shoulder subluxation, motor impairment, spasticity, and delayed rehabilitation admission are associated with shoulder-hand syndrome and upper-limb edema [4,8,25]. Despite these observations, the relationship between a threshold-based antioxidant-, nutritional-, and inflammation-related biomarker burden and upper-limb complications during inpatient stroke rehabilitation remains unclear. This gap is clinically relevant because shoulder-hand syndrome and hand edema are frequently documented during rehabilitation admission, when opportunities for prevention, monitoring, and early treatment are available [4,6,8].

A count of abnormal antioxidant-, nutritional-, and inflammation-related biomarkers could be clinically interpretable if it characterizes a clinical profile associated with clinically relevant shoulder-hand syndrome or hand edema during rehabilitation, after accounting for established neuro-musculoskeletal factors. This approach was not intended as a diagnostic, causal, or mechanistically unified model. Instead, it provides a pragmatic exposure reflecting the number of routinely measured abnormalities across antioxidant-related micronutrient status, nutritional reserve, and systemic inflammation around rehabilitation admission. Equal contribution of the four components was chosen for transparency and clinical interpretability rather than to imply equivalent biological importance. If associated with upper-limb complications and poorer short-term outcomes, this burden measure can be evaluated as an associative clinical descriptor while its incremental value over individual biomarkers is examined separately.

Therefore, this study examined whether the number of abnormal antioxidant-, nutritional-, and inflammation-related biomarkers around rehabilitation admission was associated with shoulder-hand syndrome or clinically significant hand edema during inpatient stroke rehabilitation. We also evaluated whether the same burden measure was associated with shoulder-hand syndrome and clinically significant hand edema separately, alongside Fugl–Meyer upper-extremity score at discharge, and poor upper-limb function at discharge. We hypothesized that a higher number of abnormal biomarkers would be associated with a greater likelihood of upper-limb complications and less favorable short-term upper-limb outcomes after adjusting for demographic, stroke-related, and motor variables at admission.

## 2. Materials and Methods

### 2.1. Study Design and Participants

This single-center retrospective cohort study included adults with first-ever unilateral ischemic or hemorrhagic stroke who were admitted to the Department of Rehabilitation Medicine at Kyung Hee University Hospital at Gangdong for inpatient rehabilitation between 1 January 2018 and 31 December 2025. This study was reported in accordance with the Strengthening the Reporting of Observational Studies in Epidemiology (STROBE) cohort reporting guidelines. The unit of analysis was the first inpatient rehabilitation admission after index stroke. The study protocol was reviewed and approved by the Institutional Review Board of Kyung Hee University Hospital at Gangdong (IRB number: 2026-06-056). The requirement for informed consent was waived due to the retrospective design of the study.

Patients were eligible if they were aged ≥19 years, had first-ever unilateral ischemic or hemorrhagic stroke, were admitted to inpatient rehabilitation within 60 days after stroke onset, and underwent inpatient stroke rehabilitation. Eligible patients were also required to have serum zinc, albumin, CRP, and a complete blood count with differential measured within seven days before or after Physical Medicine and Rehabilitation (PM&R) admission; Fugl–Meyer upper-extremity scores at admission and discharge; and clinical documentation regarding shoulder-hand syndrome or hand edema during the inpatient rehabilitation stay. Patients were excluded if they had recurrent stroke, bilateral stroke, lesions precluding clear upper-limb laterality, dependence in upper-limb function before stroke, missing key laboratory or upper-limb outcome data, insufficient documentation of shoulder-hand syndrome or hand edema, concomitant upper-limb fracture, amputation, severe peripheral nerve injury, severe rheumatoid or degenerative arthritis, or another upper-limb condition that interfered with the interpretation of the upper-limb functional assessment. Patients received standard inpatient stroke rehabilitation according to institutional practice, including physical and occupational therapy. Detailed therapy doses were not uniformly available for analysis. The study size was determined by including all patients who met the prespecified eligibility criteria during the study period; no formal sample size calculation was performed.

### 2.2. Data Collection and Variables

Demographic and clinical variables were extracted from electronic medical and rehabilitation records. The extracted variables included age, sex, body mass index (BMI), stroke type, onset-to-PM&R admission interval, calendar year, rehabilitation length of stay, serum zinc, albumin, CRP, neutrophil-to-lymphocyte ratio, Fugl–Meyer upper-extremity score, shoulder subluxation at admission, and spasticity at admission. Stroke type was categorized as ischemic or hemorrhagic. Shoulder subluxation at admission was considered present when subluxation of the affected shoulder was documented during the rehabilitation admission assessment. Spasticity at admission was considered present when increased tone of the affected upper limb was documented during the admission rehabilitation examination. The onset-to-PM&R admission interval was measured in days from stroke onset to PM&R admission. Rehabilitation length of stay was summarized descriptively and evaluated in a sensitivity analysis, but it was not included in the primary model because it was a post-admission variable that could be influenced by the occurrence of shoulder-hand syndrome or hand edema.

Laboratory measurements were obtained from routine clinical testing performed within seven days before or after PM&R admission. When multiple laboratory values were available within the prespecified window, the value closest to PM&R admission was used. Serum zinc was recorded in µg/dL, albumin in g/dL, C-reactive protein in mg/dL, and the neutrophil-to-lymphocyte ratio as a unitless ratio derived from the complete blood count with differential. Continuous variables were retained in the original scales for the descriptive analyses. For modeling, age was scaled per 10 years, and the admission Fugl–Meyer upper-extremity score was scaled per 10 points, as shown in the regression tables. No imputation was performed because complete information on exposure, model covariates, and outcome variables was required for inclusion in the analytical cohort. In the analytic cohort, the selected biomarker panel was obtained before PM&R admission in 205 patients (45.3%), on the day of admission in 105 (23.2%), and after admission in 143 (31.6%).

### 2.3. Outcome Measures

The primary outcome was shoulder-hand syndrome or clinically significant hand edema during inpatient rehabilitation. Because hand edema can be part of the clinical presentation of shoulder-hand syndrome, the composite outcome was intended to capture either a clinically documented diagnosis of shoulder-hand syndrome or clinically significant hand edema requiring intervention, rather than two independent disease entities [7,25]. Shoulder-hand syndrome was defined as documented shoulder or hand pain accompanied by edema of the affected hand and clinically documented as shoulder-hand syndrome or a complex regional pain syndrome-like post-stroke upper-limb condition [7,25,26]. Clinically significant hand edema was defined as documented swelling of the affected hand requiring edema-directed management, such as elevation, compression, edema massage, edema-focused range-of-motion therapy, or medication adjustment [5,27,28]. These outcomes were based on clinical documentation during the inpatient rehabilitation stay rather than prospective standardized adjudication. Outcome abstraction was performed independently by two physicians, and disagreements were resolved by consensus. The final calculated burden score was not provided to the outcome reviewers during abstraction; however, because abstraction relied on routine clinical records, masking to individual zinc, albumin, CRP, and NLR values could not be assured. For temporal analyses, the PM&R admission date, biomarker profile date, and first documented date of the primary outcome were extracted from the electronic record. Patients with the primary outcome documented on the PM&R admission date were classified as having the outcome at admission; among event cases, biomarker profiling was considered to precede the documented outcome only when the biomarker profile date was earlier than the first documented outcome date.

Supportive outcomes included shoulder-hand syndrome, clinically significant hand edema, Fugl–Meyer upper-extremity score at discharge, and poor upper-limb function at discharge [29,30,31]. Poor upper-limb function at discharge was defined as a Fugl–Meyer upper-extremity score <35. This cutoff was selected before outcome modeling as an exploratory threshold representing substantial residual upper-limb impairment at discharge. These outcomes were selected to evaluate whether the biomarker burden was associated with the primary composite clinical complication, its individual components, and short-term upper-limb functional outcomes.

### 2.4. Exposure Measure and Covariates

The primary measure was the number of abnormal antioxidant-, nutritional-, and inflammation-related biomarkers. Four routinely available biomarkers were used: serum zinc, albumin, CRP, and the neutrophil-to-lymphocyte ratio. For this study, biomarker abnormalities were operationally defined as serum zinc <70 µg/dL, albumin <3.5 g/dL, CRP ≥1.0 mg/dL, and neutrophil-to-lymphocyte ratio ≥3.0 [17,18,32,33]. These biomarker cutoffs were selected before outcome modeling based on published clinical reference ranges and commonly used inflammatory thresholds to maintain clinical interpretability [32,34,35]. The CRP ≥1.0 mg/dL and neutrophil-to-lymphocyte ratio ≥3.0 cutoffs were treated as operational thresholds for this analysis and have not been specifically validated in a post-stroke rehabilitation population. The number of abnormal antioxidant-, nutritional-, and inflammation-related biomarkers was calculated as the unweighted count of these four abnormalities and ranged from 0 to 4. Each abnormality contributed one point. Equal weighting was specified to preserve transparency and avoid outcome-driven weighting in this retrospective cohort; it was not intended to imply equivalent biological importance or a single unified biological phenotype. In the primary model, the count was analyzed as a continuous exposure, and the effect estimate was interpreted per 1-unit increase. For descriptive presentations and figures, patients were grouped as having 0, 1, 2, or 3–4 abnormal biomarkers.

The covariates in the primary model were selected a priori based on their clinical relevance and availability in the analytical dataset. The primary adjustment set included age, sex, BMI, stroke type, Fugl–Meyer upper-extremity score at admission, shoulder subluxation at admission, spasticity at admission, and onset-to-PM&R admission interval. These covariates were intended to account for demographic characteristics, stroke type, baseline upper-limb motor impairment, local mechanical or tone-related factors, and timing of rehabilitation admission.

### 2.5. Statistical Analysis

Continuous variables are summarized as means with standard deviations (SD) or medians with interquartile ranges (IQR), and categorical variables are summarized as counts and percentages. Between-group comparisons of the primary composite outcome were performed using Welch’s t-test for mean comparisons, the Mann–Whitney U test for skewed continuous variables, and Pearson’s chi-square test or Fisher’s exact test for categorical variables. Between-group differences were reported as mean, median, or risk differences with 95% confidence intervals (CIs), as appropriate. For skewed continuous variables, median differences and 95% CIs were estimated using nonparametric bootstrap resampling with 1000 resamples. Pairwise overlap among serum zinc, albumin, CRP, and the neutrophil-to-lymphocyte ratio was characterized using Spearman rank correlations. Pre-consensus agreement between the two outcome reviewers was summarized as percent agreement and Cohen’s kappa.

The primary association between the number of abnormal antioxidant-, nutritional-, and inflammation-related biomarkers and shoulder-hand syndrome or clinically significant hand edema was evaluated using multivariable logistic regression with heteroskedasticity-consistent type 3 (HC3) robust standard errors. The dependent variable was the primary composite outcome during inpatient rehabilitation. The primary model included the number of abnormal antioxidant-, nutritional-, and inflammation-related biomarkers, age, sex, BMI, stroke type, Fugl–Meyer upper-extremity score at admission, shoulder subluxation at admission, spasticity at admission, and onset-to-PM&R admission interval. Adjusted odds ratios, 95% CIs, and *p*-values are reported. Model diagnostics and descriptive performance were assessed using the area under the receiver operating characteristic curve (AUROC), Brier score, Nagelkerke R^2^, and the maximum variance inflation factor. Model diagnostics and descriptive performance indices were reported to characterize the adjusted model and were not intended to define or validate a clinical prediction tool.

Supportive analyses evaluated shoulder-hand syndrome and clinically significant hand edema separately using multivariable logistic regression with HC3 robust standard errors. Fugl–Meyer upper-extremity score at discharge was evaluated using multivariable linear regression with HC3 robust standard errors, and poor upper-limb function at discharge was evaluated using multivariable logistic regression. All supportive logistic and linear regression models used the same covariates as in the primary model. For the standardized continuous biomarker index, serum zinc and albumin were entered as inverse z scores, C-reactive protein was natural-log-transformed before standardization, and the neutrophil-to-lymphocyte ratio was standardized on its original scale. The four component z scores were averaged, and the resulting index was standardized to a standard deviation of 1 so that higher values indicated a less favorable multidomain biomarker profile. In the sex-specific zinc-cutoff analysis, low serum zinc was defined as <74 µg/dL for men and <70 µg/dL for women. Other robustness analyses included an individual biomarker model, an alternative neutrophil-to-lymphocyte ratio cutoff of ≥3.5, additional calendar-year adjustment, additional rehabilitation length-of-stay adjustment, stroke-type subgroup analyses, median quantile regression, ordinal logistic regression, categorical burden robustness analysis, influence diagnostics, and post hoc interaction analyses. Temporal sensitivity analyses used the same covariates as the primary model and considered three restrictions: (1) exclusion of patients with the primary outcome already documented at PM&R admission; (2) among event cases, retention only of those in whom the biomarker profile clearly preceded the first documented outcome date while all non-event patients were retained; and (3) restriction to patients whose biomarker profile was obtained on or before PM&R admission. To assess incremental model performance, a clinical model containing the eight prespecified clinical covariates was compared with the same model plus serum zinc, the same model plus the four-biomarker burden, and a model containing both zinc and burden. AUROC, Brier score, and Nagelkerke R^2^ were compared, and 1000 bootstrap resamples were used to estimate optimism-corrected AUROC and Brier scores. The model including both zinc and burden was also used to assess whether the burden retained an association beyond zinc after clinical adjustment. The primary inference was based on the primary model. All *p*-values were two-sided. No adjustment for multiple comparisons was applied; therefore, supportive, robustness, subgroup, influence, temporal-sensitivity, biomarker-correlation, interaction, model-comparison, and post hoc analyses were interpreted as secondary, exploratory, and hypothesis-generating. Statistical analyses were performed using R software (version 4.2.3; R Foundation for Statistical Computing, Vienna, Austria); the temporal, correlation, agreement, model-comparison, and bootstrap analyses were additionally performed using Python 3.13 (Python Software Foundation, Beaverton, OR, USA).

## 3. Results

**Figure 1:**
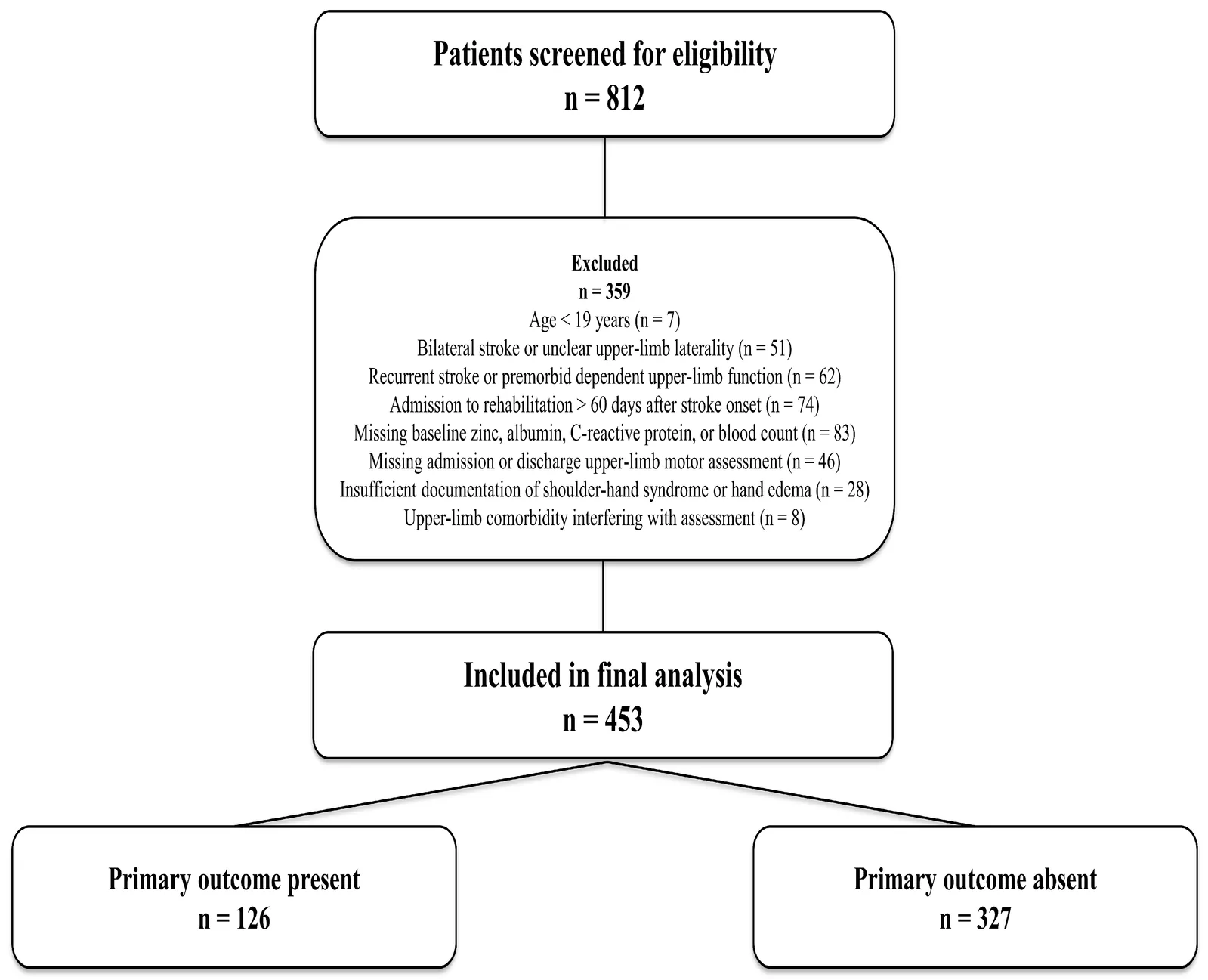
Flow of patient selection and analysis. A total of 812 patients admitted for inpatient stroke rehabilitation after a first-ever unilateral ischemic or hemorrhagic stroke were screened. After exclusions, 453 patients were included in the final analytic cohort. Among the included patients, 126 had documentation of the primary composite outcome during rehabilitation, whereas 327 had no documentation of the primary composite outcome. The primary composite outcome was defined as shoulder-hand syndrome or clinically significant hand edema during inpatient rehabilitation. PM&R, Physical Medicine and Rehabilitation.

In total, 812 patients were screened for eligibility during the study period. After excluding 359 patients, 453 patients were included in the final analytical cohort (Figure 1; Supplementary Table S2). The most frequent reasons for exclusion were missing baseline zinc, albumin, CRP, or complete blood count with differential (83 patients, 10.2% of screened patients); onset-to-PM&R admission interval >60 days (74 patients, 9.1%); recurrent stroke or dependence in upper-limb function before stroke (62 patients, 7.6%); bilateral stroke or unclear upper-limb laterality (51 patients, 6.3%); and missing admission or Fugl–Meyer upper-extremity assessment at discharge (46 patients, 5.7%). The screening-stage characteristics of the excluded patients are summarized in Supplementary Table S3. Compared with the analytic cohort, the excluded patients were older on average and had a longer onset-to-PM&R admission interval, whereas sex distribution and stroke type were similar at the screening-summary level (Supplementary Table S3). The biomarker panel was collected before PM&R admission in 205 patients (45.3%), on the admission day in 105 (23.2%), and after admission in 143 (31.6%). Twenty-eight patients had the primary composite outcome already documented at PM&R admission.

Table 1 shows baseline characteristics according to the primary composite outcome. The overall cohort had a mean age of 65.2 ± 11.2 years, 260 patients (57.4%) were male, and 331 patients (73.1%) had ischemic stroke. The mean rehabilitation length of stay was 40.4 ± 10.9 days. The primary composite outcome was documented in 126 patients, whereas 327 patients did not have the outcome. Among the 126 patients with the primary composite outcome, 23 had shoulder-hand syndrome without clinically significant hand edema, 68 had clinically significant hand edema without shoulder-hand syndrome, and 35 had both components. Patients with the primary composite outcome had a longer onset-to-PM&R admission interval than those without the outcome (30.3 ± 10.8 vs. 24.0 ± 10.7 days; mean difference [MD], 6.31; 95% CI, 4.09–8.53; *p* < 0.001). They also had lower serum zinc (68.0 ± 13.3 vs. 74.6 ± 13.7 µg/dL; MD, −6.60; 95% CI, −9.38 to −3.83; *p* < 0.001), lower albumin (3.5 ± 0.5 vs. 3.7 ± 0.5 g/dL; MD, −0.13; 95% CI, −0.24 to −0.03; *p* = 0.010), higher CRP (1.79 [0.35–3.69] vs. 0.58 [0.17–3.33] mg/dL; median difference, 1.21; 95% CI, 0.01–1.87; *p* = 0.028), and a higher neutrophil-to-lymphocyte ratio (2.74 [2.02–5.92] vs. 2.34 [1.97–4.73]; median difference, 0.40; 95% CI, 0.01–1.82; *p* = 0.024). The number of abnormal antioxidant-, nutritional-, and inflammation-related biomarkers was higher in patients with the primary composite outcome (2.1 ± 1.1 vs. 1.5 ± 1.1; MD, 0.59; 95% CI, 0.36–0.82; *p* < 0.001). The admission Fugl–Meyer upper-extremity score was lower in the outcome group (19.4 ± 14.9 vs. 29.7 ± 18.3 points; MD, −10.28; 95% CI, −13.57 to −6.99; *p* < 0.001). Shoulder subluxation at admission and spasticity at admission were also more frequent in the outcome group (31.7% vs. 8.9%; risk difference [RD], 22.9 percentage points; 95% CI, 14.2–31.6; *p* < 0.001; and 37.3% vs. 22.6%; RD, 14.7 percentage points; 95% CI, 5.1–24.3; *p* = 0.002, respectively).

**Figure 2:**
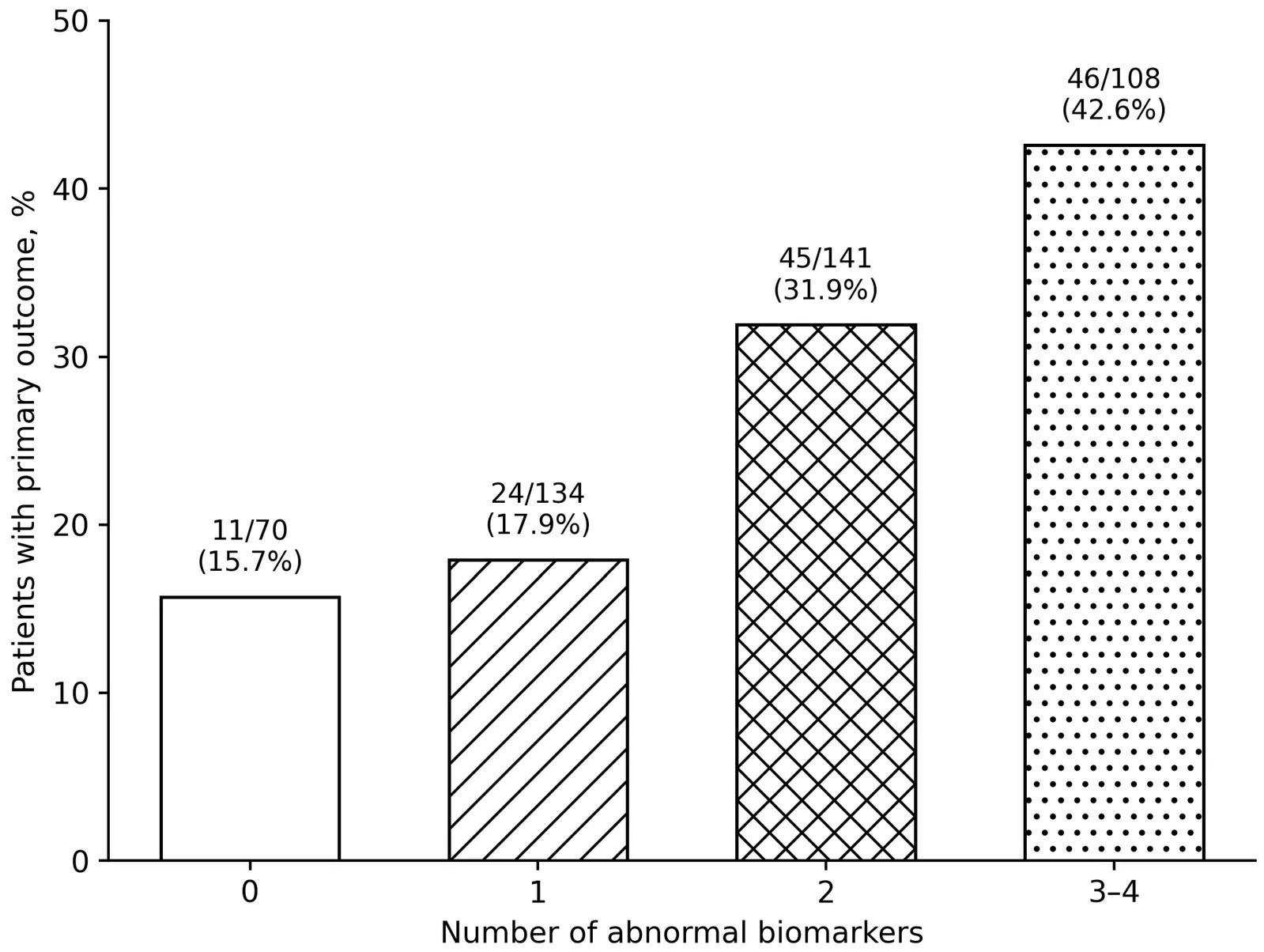
Documented primary composite outcome according to the number of abnormal antioxidant-, nutritional-, and inflammation-related biomarkers. Bars show the unadjusted proportions of patients with the primary composite outcome in each burden category. The primary composite outcome was documented in 15.7% (11/70) of patients with 0 abnormal biomarkers, 17.9% (24/134) with 1 abnormal biomarker, 31.9% (45/141) with 2 abnormal biomarkers, and 42.6% (46/108) with 3–4 abnormal biomarkers. Abnormal biomarkers were defined as serum zinc <70 µg/dL, albumin <3.5 g/dL, C-reactive protein ≥1.0 mg/dL, and neutrophil-to-lymphocyte ratio ≥3.0.

The distribution of antioxidant-, nutritional-, and inflammation-related biomarkers is shown in Supplementary Table S1. Serum zinc <70 µg/dL was present in 198 patients (43.7%), albumin <3.5 g/dL in 187 patients (41.3%), CRP ≥1.0 mg/dL in 207 patients (45.7%), and neutrophil-to-lymphocyte ratio ≥3.0 in 175 patients (38.6%). The burden distribution was as follows: 70 patients (15.5%) had 0 abnormal biomarkers; 134 (29.6%) had 1; 141 (31.1%) had 2; 81 (17.9%) had 3; and 27 (6.0%) had 4. The primary composite outcome increased across the planned burden categories: 11 of 70 patients (15.7%) had 0 abnormal biomarkers; 24 of 134 (17.9%) had 1; 45 of 141 (31.9%) had 2; and 46 of 108 (42.6%) had 3–4 abnormal biomarkers (Figure 2; Supplementary Table S6). In the categorical burden robustness analysis using 0 abnormal biomarkers as the reference, adjusted ORs were 1.05 (95% CI, 0.44–2.49) for 1 abnormal biomarker, 2.02 (95% CI, 0.90–4.51) for 2, 2.06 (95% CI, 0.88–4.81) for 3, and 4.42 (95% CI, 1.42–13.77) for 4. Although the category-specific estimates were imprecise, there was no statistical evidence of departure from linearity (*p* = 0.589).

**Figure 3:**
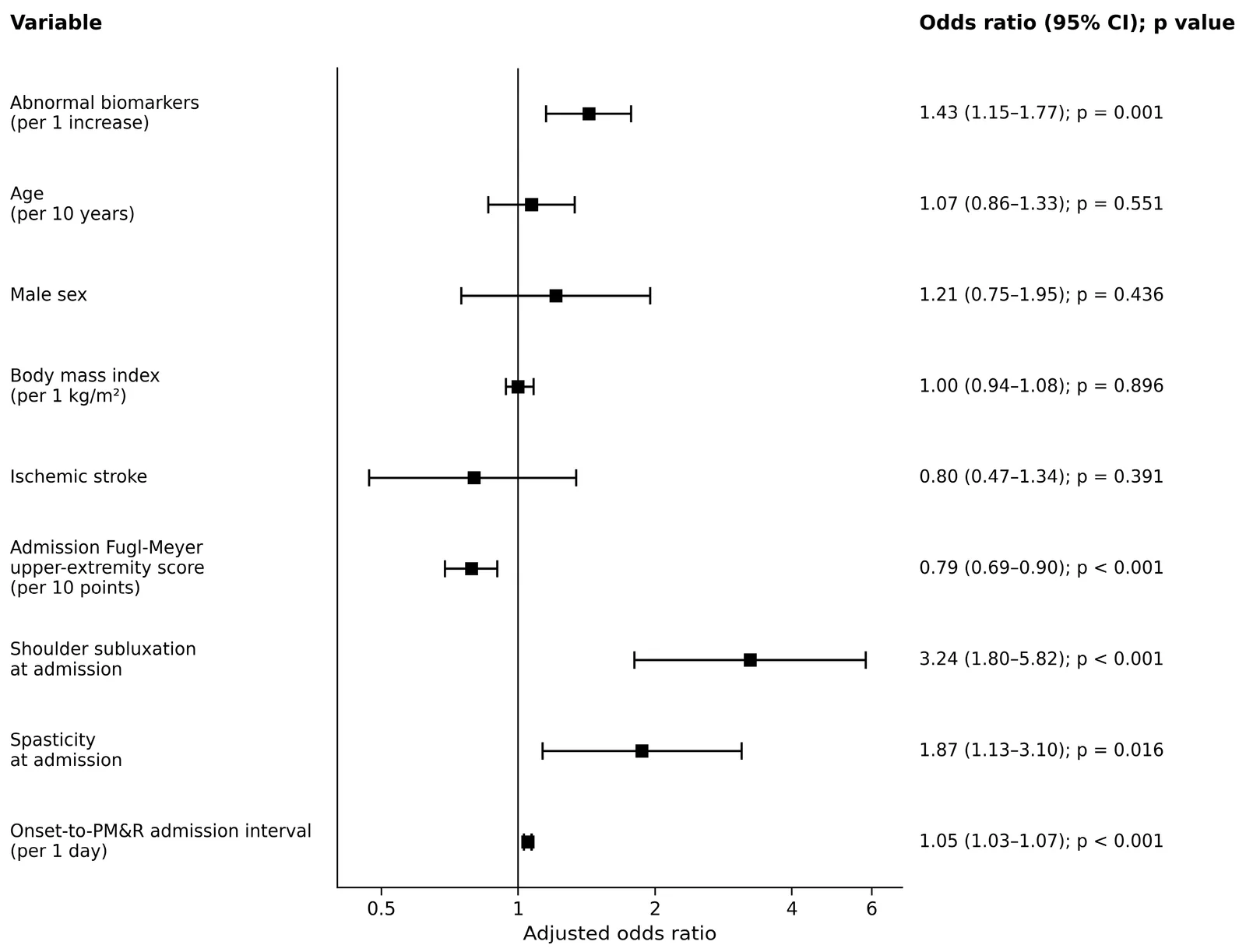
Multivariable logistic regression analysis for the primary composite outcome. Squares indicate adjusted odds ratios, and horizontal lines indicate 95% confidence intervals. The model included the number of abnormal antioxidant-, nutritional-, and inflammation-related biomarkers, age, sex, body mass index, stroke type, admission Fugl–Meyer upper-extremity score, shoulder subluxation at admission, spasticity at admission, and onset-to-PM&R admission interval. Estimates are presented on the odds-ratio scale, with 1 indicating the null value. CI, confidence interval; PM&R, Physical Medicine and Rehabilitation.

Table 2 presents the primary multivariable logistic regression model. Each 1-unit increase in the number of abnormal antioxidant-, nutritional-, and inflammation-related biomarkers was associated with higher odds of shoulder-hand syndrome or clinically significant hand edema (OR, 1.43; 95% CI, 1.15–1.77; *p* = 0.001). The admission Fugl–Meyer upper-extremity score was inversely associated with the primary composite outcome (OR per 10-point increase, 0.79; 95% CI, 0.69–0.90; *p* < 0.001). Shoulder subluxation at admission (OR, 3.24; 95% CI, 1.80–5.82; *p* < 0.001), spasticity at admission (OR, 1.87; 95% CI, 1.13–3.10; *p* = 0.016), and onset-to-PM&R admission interval (OR per 1-day increase, 1.05; 95% CI, 1.03–1.07; *p* < 0.001) were also associated with higher odds of the primary composite outcome. Age, male sex, BMI, and ischemic stroke were not statistically significant in the primary model. The model had an AUROC of 0.78, a Brier score of 0.160, a Nagelkerke R^2^ of 0.28, and a maximum VIF of 1.14. Figure 3 shows the adjusted ORs and 95% CIs for the primary model.

Odds ratios were estimated using multivariable logistic regression with HC3 robust standard errors. The dependent variable was shoulder-hand syndrome or clinically significant hand edema during inpatient rehabilitation. The model was adjusted for age, sex, BMI, stroke type, FMA-UE score at admission, shoulder subluxation, spasticity, and onset-to-PM&R admission interval. The model diagnostics and descriptive performance values were obtained using the same primary model. AUROC, area under the receiver operating characteristic curve; BMI, body mass index; CI, confidence interval; FMA-UE, Fugl–Meyer assessment of the upper extremity; OR, odds ratio; PM&R, Physical Medicine and Rehabilitation; VIF, variance inflation factor. Model diagnostics and descriptive performance values were used to describe model discrimination and fit and were not intended to define or validate a clinical prediction tool.

The supportive analyses are summarized in Table 3. Each 1-unit increase in the number of abnormal antioxidant-, nutritional-, and inflammation-related biomarkers was associated with higher odds of shoulder-hand syndrome (adjusted OR, 1.43; 95% CI, 1.06–1.92; *p* = 0.018) and clinically significant hand edema (adjusted OR, 1.49; 95% CI, 1.18–1.87; *p* < 0.001). A higher burden was also associated with a lower Fugl–Meyer upper-extremity score at discharge (mean difference, −1.90; 95% CI, −2.87 to −0.92; *p* < 0.001). In the model of poor upper-limb function at discharge, defined as a Fugl–Meyer upper-extremity score at discharge <35, the adjusted OR was 1.37 (95% CI, 1.09–1.72; *p* = 0.006). These supportive analyses were exploratory and were not treated as independent confirmatory evidence.

Supplementary Table S4 presents the individual biomarker model. Higher serum zinc was associated with lower odds of the primary composite outcome (OR per 1 µg/dL increase, 0.97; 95% CI, 0.96–0.99; *p* = 0.003), whereas albumin, natural-log-transformed CRP, and neutrophil-to-lymphocyte ratio were not statistically significant in the same model. Supplementary Table S5 shows the previously specified robustness, subgroup, influence, and post hoc analyses. Directionally consistent estimates were observed for the standardized continuous biomarker index (adjusted OR, 1.52; 95% CI, 1.20–1.93; *p* < 0.001), alternative NLR cutoff, sex-specific zinc cutoffs, calendar-year adjustment, rehabilitation length-of-stay adjustment, and stroke-type subgroup analyses. After excluding 25 observations with Cook’s distance >4/N, the adjusted OR increased from 1.43 to 1.84 (95% CI, 1.44–2.34; *p* < 0.001), indicating that the direction was unchanged but the effect magnitude was sensitive to influential observations. The 25 influential observations included 22 (88.0%) patients with the primary composite outcome; 16 (64.0%) had 0–1 abnormal biomarkers, and their mean burden was 1.2 compared with 1.7 among the remaining 428 patients. Pairwise Spearman correlations among the four biomarkers were weak, with coefficients ranging from −0.221 to 0.161 (Supplementary Table S7), indicating only partial overlap among components. Pre-consensus reviewer agreement was 94.0% for the primary composite outcome (Cohen’s κ = 0.855), 96.9% for shoulder-hand syndrome (κ = 0.862), and 95.6% for clinically significant hand edema (κ = 0.874). In model-performance comparisons, the clinical model alone had an AUROC of 0.763, Brier score of 0.164, and Nagelkerke R^2^ of 0.248; the clinical-plus-zinc model had corresponding values of 0.778, 0.159, and 0.279; and the clinical-plus-burden model had values of 0.776, 0.160, and 0.275 (Supplementary Table S8). Bootstrap optimism-corrected AUROC/Brier values were 0.744/0.171, 0.758/0.167, and 0.756/0.168, respectively. The clinical-plus-zinc-plus-burden model had an apparent AUROC of 0.781, Brier score of 0.158, and Nagelkerke R^2^ of 0.285, with optimism-corrected AUROC/Brier values of 0.758/0.167; these corrected performance estimates were essentially unchanged from the clinical-plus-zinc model. When zinc and burden were both added to the clinical model, the burden estimate was attenuated (adjusted OR, 1.22; 95% CI, 0.95–1.58; *p* = 0.125), indicating no clear incremental association beyond zinc. Temporal sensitivity analyses were directionally consistent (Supplementary Table S9). After excluding the 28 patients with the primary outcome already documented at PM&R admission, 425 patients remained (98 events); biomarker profiling preceded the documented outcome in all 98 subsequent event cases, and the adjusted OR was 1.42 (95% CI, 1.12–1.79; *p* = 0.004). When event cases were restricted to those with biomarker profiling clearly preceding the documented outcome while all non-event patients were retained (*N* = 442), the adjusted OR was 1.41 (95% CI, 1.13–1.76; *p* = 0.003). Restriction to 310 patients with biomarker profiling on or before PM&R admission yielded an adjusted OR of 1.43 (95% CI, 1.11–1.85; *p* = 0.006).

## 4. Discussion

In this single-center retrospective cohort of adults undergoing inpatient rehabilitation after a first-ever unilateral stroke, a higher number of abnormal antioxidant-, nutritional-, and inflammation-related biomarkers was associated with shoulder-hand syndrome or clinically significant hand edema during rehabilitation. This association persisted after adjustment for demographic characteristics, stroke type, admission Fugl–Meyer upper-extremity score, shoulder subluxation at admission, spasticity at admission, and onset-to-PM&R admission interval and remained directionally consistent in temporally restricted sensitivity analyses. The direction was also similar when shoulder-hand syndrome and clinically significant hand edema were evaluated separately. A higher burden was additionally associated with a lower baseline-adjusted Fugl–Meyer upper-extremity score at discharge and higher odds of poor upper-limb function at discharge, although these supportive outcomes are exploratory. These findings characterize an associative clinical profile spanning antioxidant-related micronutrient status, nutritional reserve, and inflammation rather than a unified biological phenotype or evidence of causality.

The primary finding should be interpreted as an association rather than evidence of causality. Shoulder-hand syndrome and hand edema after stroke are multifactorial conditions [7,9,25]. Motor weakness, dependent positioning, shoulder subluxation, altered tone, autonomic dysfunction, pain sensitization, and delayed rehabilitation may all contribute to the clinical presentation [7,8,9,36]. In the present analysis, the Fugl–Meyer upper-extremity score, shoulder subluxation at admission, spasticity at admission, and onset-to-PM&R admission interval were all associated with the primary composite outcome, which is consistent with a neuro-musculoskeletal framework [4,7,8]. The association observed after multivariable adjustment suggests that a systemic clinical state may provide associative information beyond measured local motor and mechanical factors.

Several plausible mechanisms may help explain this observed pattern [37,38]. Lower serum zinc is biologically relevant to antioxidant defense, immune regulation, and tissue repair [15,39,40]. Lower albumin may reflect nutritional reserve and antioxidant capacity but is also influenced by inflammation and systemic illness [16,33,41]. Elevated CRP levels and neutrophil-to-lymphocyte ratios may reflect systemic inflammatory activation, which can influence vascular permeability, edema formation, nociceptive sensitivity, and recovery potential [17,42,43]. However, these pathways are not mutually exclusive. The burden count used in this study was intended to capture the accumulation of routine laboratory abnormalities rather than isolate a single pathway. This approach may be clinically interpretable, because patients may show overlapping nutritional, antioxidant, and inflammatory abnormalities during the early rehabilitation phase [33,44,45]. Importantly, the four biomarkers reflect distinct and only partly overlapping processes; the burden count therefore summarizes co-occurring abnormalities and should not be interpreted as evidence of a single biological mechanism.

The individual biomarker model suggested that serum zinc had the most consistent signal after simultaneous adjustment for albumin, natural-log-transformed CRP, and the neutrophil-to-lymphocyte ratio. However, this finding should be interpreted with caution because serum zinc levels can be influenced by acute inflammation, nutritional status, timing of blood sampling, and concurrent medical conditions [46,47,48]. Nevertheless, zinc is directly relevant to antioxidant biology and immune regulation, and these results provide a rationale for further investigation of zinc status during stroke rehabilitation [39,49,50]. Albumin, CRP, and neutrophil-to-lymphocyte ratio showed directions compatible with an unfavorable clinical profile; however, their individual effects were less precise in the simultaneous model. The model-comparison analysis did not demonstrate clear incremental benefit of the burden beyond serum zinc: apparent and bootstrap-corrected performance were similar for the clinical-plus-zinc and clinical-plus-burden models, and adding the burden to the clinical-plus-zinc model left the optimism-corrected AUROC/Brier values unchanged at 0.758/0.167 while the burden estimate was attenuated. Thus, the present data support further comparison of the multidomain burden with individual biomarkers rather than establishing preference for the composite measure.

The baseline-adjusted Fugl–Meyer upper-extremity score at discharge was treated as the principal continuous functional outcome and should be interpreted as an exploratory association with short-term upper-limb function rather than evidence of impaired biological recovery. Although the estimated difference per 1-unit increase in burden was modest, patients with 3–4 abnormal biomarkers had a higher frequency of the primary composite outcome and less favorable descriptive upper-limb outcomes than those with fewer abnormalities. These results suggest that laboratory profiles around rehabilitation admission may help characterize a clinical profile associated with edema, pain-related upper-limb complications, and less favorable short-term motor outcomes, but they do not establish prospective predictive utility.

From a clinical perspective, the number of abnormal antioxidant-, nutritional-, and inflammation-related biomarkers should not be viewed as a validated prediction, screening, diagnostic, or treatment-selection score. Rather, the observed association provides a rationale for future prospective evaluation alongside careful assessment of the affected upper limb, edema management, attention to nutritional status, and coordinated rehabilitation care [1,5,51]. Prospective studies should determine whether structured assessment of zinc status, nutritional indicators, and inflammatory markers adds clinically useful information when biomarker measurements clearly precede upper-limb complications. Interventional studies are needed before any conclusion can be drawn about whether the correction of specific abnormalities changes the frequency or severity of shoulder-hand syndrome, hand edema, or short-term upper-limb impairment.

This study has several strengths. The cohort was defined using a clear rehabilitation admission window after first-ever unilateral stroke, and the primary outcome reflected clinically relevant upper-limb complications documented during inpatient rehabilitation. The exposure used routinely available laboratory markers, which may increase clinical interpretability. The primary model was adjusted for major neuro-musculoskeletal factors, including Fugl–Meyer upper-extremity score, shoulder subluxation, spasticity, and timing of rehabilitation admission. Multiple supportive analyses were performed, including alternative biomarker definitions, a standardized continuous index, calendar-year adjustment, rehabilitation length-of-stay adjustment, stroke-type subgroup analyses, and influence diagnostics. Additional analyses included temporal sensitivity analyses, inter-rater agreement, biomarker correlation analysis, and bootstrap-corrected model-performance comparisons.

This study has several limitations. First, although retrospective data review allowed restricted sensitivity analyses, the retrospective design does not establish true temporal precedence in all patients: 31.6% of biomarker profiles were obtained after PM&R admission and 28 patients already had the primary outcome at admission. The available outcome dates represent the first clinical documentation rather than prospectively ascertained symptom-onset dates; therefore, reverse causation or a concurrent biological state cannot be excluded even when biomarker profiling preceded the documented outcome. The temporally restricted estimates were directionally consistent. In addition, excluding 25 influential observations increased the adjusted OR from 1.43 to 1.84, indicating sensitivity in effect magnitude despite an unchanged direction. Second, the single-center design limits generalizability. Third, exclusions for missing laboratory, Fugl–Meyer, or outcome-documentation data may have introduced selection bias. Fourth, shoulder-hand syndrome and clinically significant hand edema were defined using clinical documentation rather than prospective standardized adjudication; therefore, underrecognition, practice-pattern variation, or misclassification is possible. Standardized diagnostic criteria for complex regional pain syndrome and standardized edema grading were not uniformly available in the retrospective records [52,53]. Pre-consensus inter-rater agreement for the primary composite outcome was high (94.0%; Cohen’s κ = 0.855), but outcome ascertainment remains susceptible to underrecognition, practice-pattern variation, and retrospective misclassification. In addition, shoulder-hand syndrome and clinically significant hand edema are overlapping clinical constructs because hand edema can be a part of shoulder-hand syndrome [7,25]. Therefore, the primary composite outcome should be interpreted as a pragmatic rehabilitation complication rather than as a composite of fully independent components. Fifth, serum zinc, albumin, CRP, and the neutrophil-to-lymphocyte ratio, rather than direct measures of oxidative stress, are indirect indicators spanning antioxidant-related micronutrient status, nutritional reserve, and inflammation [17,18,33,47]. The burden score assigned equal weights to each biomarker abnormality and relied on dichotomized cutoffs, which may have reduced information from continuous biomarker distributions; equal weighting should not be interpreted as biological equivalence. Sixth, serum zinc and albumin may vary with inflammatory state, and CRP and the neutrophil-to-lymphocyte ratio may be affected by infection, medications, comorbidities, and acute inflammatory conditions that were not fully captured [17,18]. Seventh, potentially relevant variables including infection status, detailed systemic comorbidities and treatments, organ dysfunction, lesion location or volume, baseline global neurological severity, detailed pain treatment, nutritional intervention, and rehabilitation intensity were not available with sufficient completeness for additional multivariable adjustment in the analytic dataset; residual confounding therefore remains possible. Eighth, the Fugl–Meyer upper-extremity score represents short-term upper-limb motor impairment and does not fully describe long-term hand function [30]. Finally, supportive, robustness, subgroup, influence, temporal-sensitivity, biomarker-correlation, model-comparison, interaction, and post hoc analyses were not adjusted for multiple comparisons and should be interpreted as exploratory and hypothesis-generating.

## 5. Conclusions

A higher number of abnormal antioxidant-, nutritional-, and inflammation-related biomarkers around rehabilitation admission was associated with shoulder-hand syndrome or clinically significant hand edema during inpatient stroke rehabilitation. The burden measure was also associated with a lower baseline-adjusted Fugl–Meyer upper-extremity score at discharge and higher odds of poor upper-limb function at discharge. Temporal sensitivity analyses were directionally consistent, but model comparisons showed only small changes in performance metrics over clinical covariates and no clear incremental association of the burden beyond serum zinc when both were included. These findings characterize an associative clinical profile rather than validate the burden measure for prediction, screening, diagnosis, or treatment selection. Prospective studies with standardized outcome ascertainment and biomarker measurements clearly preceding upper-limb complications are required to establish clinical utility.

## Figures and Tables

**Table 1:** Baseline characteristics according to shoulder-hand syndrome or clinically significant hand edema.

Characteristic	Overall (*N* = 453)	No Shoulder-Hand Syndrome or Clinically Significant Hand Edema (*N* = 327)	Shoulder-Hand Syndrome or Clinically Significant Hand Edema (*N* = 126)	Between-Group Difference (95% CI)	*p* Value
Age, years	65.2 ± 11.2	64.6 ± 11.2	66.8 ± 11.0	MD 2.22 (−0.07 to 4.51)	0.058
Male, sex	260 (57.4)	187 (57.2)	73 (57.9)	RD 0.7 (−9.4 to 10.9)	0.885
BMI, kg/m^2^	24.2 ± 3.2	24.3 ± 3.2	24.0 ± 3.1	MD −0.30 (−0.94 to 0.35)	0.365
Ischemic stroke	331 (73.1)	246 (75.2)	85 (67.5)	RD −7.8 (−17.2 to 1.7)	0.095
Onset-to-PM&R admission interval, days	25.7 ± 11.1	24.0 ± 10.7	30.3 ± 10.8	MD 6.31 (4.09 to 8.53)	<0.001
Serum zinc, µg/dL	72.8 ± 13.9	74.6 ± 13.7	68.0 ± 13.3	MD −6.60 (−9.38 to −3.83)	<0.001
Albumin, g/dL	3.6 ± 0.5	3.7 ± 0.5	3.5 ± 0.5	MD −0.13 (−0.24 to −0.03)	0.010
C-reactive protein, mg/dL	0.67 [0.19–3.42]	0.58 [0.17–3.33]	1.79 [0.35–3.69]	Median difference 1.21 (0.01 to 1.87)	0.028
Neutrophil-to-lymphocyte ratio	2.45 [1.99–5.00]	2.34 [1.97–4.73]	2.74 [2.02–5.92]	Median difference 0.40 (0.01 to 1.82)	0.024
Number of abnormal antioxidant-, nutritional-, and inflammation-related biomarkers	1.7 ± 1.1	1.5 ± 1.1	2.1 ± 1.1	MD 0.59 (0.36 to 0.82)	<0.001
Admission Fugl–Meyer upper-extremity score, points	26.8 ± 18.0	29.7 ± 18.3	19.4 ± 14.9	MD −10.28 (−13.57 to −6.99)	<0.001
Shoulder subluxation at admission	69 (15.2)	29 (8.9)	40 (31.7)	RD 22.9 (14.2 to 31.6)	<0.001
Spasticity at admission	121 (26.7)	74 (22.6)	47 (37.3)	RD 14.7 (5.1 to 24.3)	0.002

Values are mean ± SD, median [IQR], or *n* (%). Difference is calculated as the outcome group minus the no-outcome group. MD, mean difference; median difference, difference in group medians; RD, risk difference in percentage points. *p*-values were obtained using Welch’s t-test for mean comparisons, the Mann–Whitney *U* test for skewed variables, and Pearson’s chi-square test or Fisher’s exact test for categorical variables. BMI, body mass index; CI, confidence interval; IQR, interquartile range; PM&R, Physical Medicine and Rehabilitation; SD, standard deviation.

**Table 2:** Multivariable logistic regression for shoulder-hand syndrome or clinically significant hand edema.

Variable or Model Measure	Estimate or Value	95% CI	*p* Value
Number of abnormal antioxidant-, nutritional-, and inflammation-related biomarkers (per 1 increase)	1.43	1.15–1.77	0.001
Age (per 10 years)	1.07	0.86–1.33	0.551
Male sex (vs. female)	1.21	0.75–1.95	0.436
BMI (per 1 kg/m^2^)	1.00	0.94–1.08	0.896
Ischemic stroke (vs. hemorrhagic stroke)	0.80	0.47–1.34	0.391
Admission Fugl–Meyer upper-extremity score (per 10 points)	0.79	0.69–0.90	<0.001
Shoulder subluxation at admission (yes vs. no)	3.24	1.80–5.82	<0.001
Spasticity at admission (yes vs. no)	1.87	1.13–3.10	0.016
Onset-to-PM&R admission interval (per 1 day)	1.05	1.03–1.07	<0.001
Model diagnostics and descriptive performance			
AUROC	0.78		
Brier score	0.160		
Nagelkerke R^2^	0.28		
Maximum VIF	1.14		

**Table 3:** Supportive Analyses for Secondary Outcomes.

Outcome or Model	Effect Estimate	Estimate	95% CI	*p* Value	Model Diagnostics/Additional Information
Shoulder-hand syndrome	Adjusted OR	1.43	1.06–1.92	0.018	AUROC 0.75; Brier 0.098; Nagelkerke R^2^ 0.18
Clinically significant hand edema	Adjusted OR	1.49	1.18–1.87	<0.001	AUROC 0.77; Brier 0.148; Nagelkerke R^2^ 0.24
Fugl–Meyer upper-extremity score at discharge	Mean difference	−1.90	−2.87 to −0.92	<0.001	adjusted R^2^ 0.59; RMSE 10.7
Poor upper-limb function at discharge	Adjusted OR	1.37	1.09–1.72	0.006	AUROC 0.85; Brier 0.160; Nagelkerke R^2^ 0.45

Unless otherwise specified, the estimates represent the effect per 1-unit increase in the number of abnormal antioxidant-, nutritional-, and inflammation-related biomarkers. Logistic regression estimates were adjusted ORs and the linear regression estimate was the mean difference in FMA-UE score at discharge. All logistic and linear regression models used HC3 robust standard errors and the same covariates as those used in the primary model. AUROC, area under the receiver operating characteristic curve; CI, confidence interval; FMA-UE, Fugl–Meyer assessment of the upper extremity; OR, odds ratio; RMSE, root mean square error.

## Data Availability

The data presented in this study are available from the corresponding author upon reasonable request. The data are not publicly available because they contain clinical information that is subject to patient privacy, institutional, and ethical restrictions.

## Supplementary Materials

The following supporting information can be downloaded at https://www.mdpi.com/article/10.3390/antiox15091173/s1, Table S1, Definition and Distribution of Antioxidant-, Nutritional-, and Inflammation-Related Biomarkers; Table S2, Screening Log and Exclusion Details; Table S3, Screening-Stage Characteristics of Excluded Patients; Table S4, Individual Biomarker Model for Shoulder-Hand Syndrome or Clinically Significant Hand Edema; Table S5, Sensitivity and Post-Hoc Analyses; Table S6, Outcome Distribution According to Biomarker Burden Level; Table S7, Pairwise Spearman Correlations among Biomarkers; Table S8, Incremental Model Performance and Bootstrap Internal Validation; Table S9, Temporal Sensitivity Analyses.

## Abbreviations

The following abbreviations are used in this manuscript: AUROC, area under the receiver operating characteristic curve; BMI, body mass index; CI, confidence interval; CRP, C-reactive protein; FMA-UE, Fugl-Meyer Assessment of the Upper Extremity; HC3, heteroskedasticity-consistent type 3; IQR, interquartile range; MD, mean difference; NLR, neutrophil-to-lymphocyte ratio; OR, odds ratio; PM&R, Physical Medicine and Rehabilitation; RD, risk difference; RMSE, root mean square error; SD, standard deviation; VIF, variance inflation factor.
