# Supplementary material for: A Higher Burden of Abnormal Antioxidant-, Nutritional-, and Inflammation-Related Biomarkers Is Associated with Shoulder-Hand Syndrome, Hand Edema, and Short-Term Upper-Limb Outcomes During Stroke Rehabilitation
Source: Antioxidants (Basel). 2026 Sep 16;15(9):1173. doi: 10.3390/antiox15091173 (PMC13603648; doi:10.3390/antiox15091173)
Supplement: Supplementary file 1 [file antioxidants-15-01173-s001.zip › antioxidants-4469388-supplementary.pdf]

Supplementary Tables

Table S1. Definition and Distribution of Antioxidant-, Nutritional-, and Inflammation-Related Biomarkers

Biomarker or burden level	Summary value	Abnormal definition or category	n (%)
Serum zinc	72.8 ± 13.9 µg/dL	<70 µg/dL	198 (43.7)
Albumin	3.6 ± 0.5 g/dL	<3.5 g/dL	187 (41.3)
C-reactive protein	0.67 [0.19–3.42] mg/dL	≥1.0 mg/dL	207 (45.7)
Neutrophil-to-lymphocyte ratio	2.45 [1.99–5.00]	≥3.0	175 (38.6)
0 abnormal biomarkers		Burden distribution	70 (15.5)
1 abnormal biomarker		Burden distribution	134 (29.6)
2 abnormal biomarkers		Burden distribution	141 (31.1)
3 abnormal biomarkers		Burden distribution	81 (17.9)
4 abnormal biomarkers		Burden distribution	27 (6.0)

Values are mean ± SD, median [IQR], or n (%). The number of abnormal antioxidant-, nutritional-, and inflammation-related biomarkers was calculated as the count of serum zinc <70 µg/dL, albumin <3.5 g/dL, CRP ≥1.0 mg/dL, and NLR ≥3.0. CRP, C-reactive protein; IQR, interquartile range; NLR, neutrophil-to-lymphocyte ratio; SD, standard deviation.

Table S2. Screening Log and Exclusion Details

Screening step or exclusion criterion	Patients, n	% of screened
Screened for eligibility	812	100.0
Excluded	359	44.2
Age <19 years	7	0.9
Bilateral stroke or lesion with unclear upper-limb laterality	51	6.3
Recurrent stroke or premorbid dependent upper-limb function	62	7.6
Onset-to-PM&R admission interval >60 days	74	9.1
Missing baseline zinc, albumin, CRP, or CBC with differential count	83	10.2
Missing admission or discharge Fugl-Meyer upper-extremity assessment	46	5.7
Insufficient documentation of shoulder-hand syndrome or hand edema	28	3.4
Concomitant upper-limb fracture, peripheral nerve injury, severe arthritis, or amputation interfering with assessment	8	1.0
Included in final analytic cohort	453	55.8

The percentages were calculated using the total screened population as the denominator (N = 812). The exclusion categories were treated as mutually exclusive in screening logs. CBC, complete blood count; CRP, C-reactive protein; FMA-UE, Fugl-Meyer assessment of the upper extremity; PM&R, Physical Medicine and Rehabilitation.

Table S3. Screening-Stage Characteristics of Excluded Patients

Screening-stage characteristic	Value
Excluded patients	359
Age, years	67.9 ± 12.8
Male sex	203 (56.5)
BMI, kg/m²	23.4 ± 3.6
Ischemic stroke	261 (72.7)
Hemorrhagic stroke	98 (27.3)
Onset-to-PM&R admission interval, days	34.8 ± 31.6
Baseline zinc available	221 (61.6)
Baseline Fugl-Meyer upper-extremity score available	285 (79.4)

Values are mean ± SD or n (%). These screening-stage summary data describe the nonincluded population and were not included in the patient-level analyses. BMI, body mass index; FMA-UE, Fugl-Meyer assessment of the upper extremity; PM&R, Physical Medicine and Rehabilitation; SD, standard deviation.

Table S4. Individual Biomarker Model for Shoulder-Hand Syndrome or Clinically Significant Hand Edema

Predictor	Unit	Adjusted odds ratio	95% CI	p value
Serum zinc	per 1 μg/dL increase	0.97	0.96–0.99	0.003
Albumin	per 1 g/dL increase	0.79	0.48–1.28	0.335
Natural-log-transformed C-reactive protein	per 1-unit increase in natural-log scale	1.09	0.95–1.27	0.229
Neutrophil-to-lymphocyte ratio	per 1-unit increase	1.03	0.93–1.15	0.578

The model simultaneously included serum zinc, albumin, natural-log-transformed CRP, and NLR, and was adjusted for the same covariates as the primary model. Model diagnostics: AUROC 0.78; Brier score 0.158; Nagelkerke R^2 0.29. CI, confidence interval; CRP, C-reactive protein; NLR, neutrophil-to-lymphocyte ratio; OR, odds ratio.

Table S5. Sensitivity and Post-Hoc Analyses

Analysis	Outcome or model	Effect estimate	Estimate	95% CI	p value	Notes
Robustness analysis	Primary composite outcome; standardized continuous index (per 1 SD)	Adjusted OR	1.52	1.20–1.93	<0.001	AUROC 0.78; Brier 0.160; Nagelkerke R^2 0.28
Robustness analysis	Primary composite outcome; NLR cutoff ≥3.5	Adjusted OR	1.40	1.13–1.74	0.002	AUROC 0.78; Brier 0.161; Nagelkerke R^2 0.27
Robustness analysis	Primary composite outcome; sex-specific zinc cutoff	Adjusted OR	1.41	1.14–1.75	0.002	AUROC 0.78; Brier 0.160; Nagelkerke R^2 0.27
Robustness analysis	Primary composite outcome; additional calendar-year adjustment	Adjusted OR	1.42	1.15–1.77	0.001	AUROC 0.78; Brier 0.160; Nagelkerke R^2 0.28
Robustness analysis	Primary composite outcome; additional rehabilitation length-of-stay adjustment	Adjusted OR	1.42	1.15–1.77	0.001	AUROC 0.78; Brier 0.160; Nagelkerke R^2 0.28
Subgroup analysis	Ischemic stroke subgroup	Adjusted OR	1.37	1.06–1.78	0.017	N = 331; outcome events = 85; AUROC 0.79; Brier 0.149; Nagelkerke R^2 0.30
Subgroup analysis	Hemorrhagic stroke subgroup	Adjusted OR	1.52	0.99–2.32	0.055	N = 122; outcome events = 41; AUROC 0.75; Brier 0.187; Nagelkerke R^2 0.22
Robustness analysis	Discharge Fugl-Meyer upper-extremity score; median quantile regression	Median difference	-1.98	-3.10 to -0.85	<0.001	
Robustness analysis	Discharge Fugl-Meyer upper-	OR for worse tertile	1.41	1.16–1.72	<0.001	

Analysis	Outcome or model	Effect estimate	Estimate	95% CI	p value	Notes
	extremity tertile; ordinal logistic regression					
Categorical burden robustness	Primary composite outcome	p for departure from linearity			0.589	Categorical burden model compared with the continuous burden model to assess departure from linearity
Influence diagnostics	Primary model after excluding Cook's distance >4/N observations	Adjusted OR	1.84	1.44–2.34	<0.001	25 observations excluded; AUROC 0.84; Brier 0.127; Nagelkerke R^2 0.40
Post-hoc interaction	Primary composite outcome	burden × shoulder subluxation at admission	1.44	0.82–2.53	0.206	Interaction term odds ratio
Post-hoc interaction	Primary composite outcome	burden × spasticity at admission	0.99	0.61–1.62	0.972	Interaction term odds ratio

Unless otherwise specified, the sensitivity analyses used the primary composite outcome and the same adjustment set as the primary model. The standardized continuous biomarker index was calculated by averaging four component z scores: inverse-standardized serum zinc, inverse-standardized albumin, standardized natural-log-transformed C-reactive protein, and standardized neutrophil-to-lymphocyte ratio on its original scale; the average was then standardized to a standard deviation of 1. This index was used as a robustness analysis and did not assume that the four biomarkers represented a single biological pathway. For the sex-specific zinc-cutoff analysis, low serum zinc was defined as <74 μg/dL for men and <70 μg/dL for women. Interaction analyses were performed using the centered burden count. CI, confidence interval; FMA-UE, Fugl-Meyer assessment of the upper extremity; NLR, neutrophil-to-lymphocyte ratio; OR, odds ratio; PM&R, Physical Medicine and Rehabilitation.

Table S6. Outcome Distribution According to Biomarker Burden Level

Number of abnormal biomarkers	Patients, n (%)	Primary composite outcome, n (%)	Shoulder-hand syndrome, n (%)	Clinically significant hand edema, n (%)	Poor discharge upper-limb function, n (%)	Discharge FMA-UE score, mean ± SD
0	70 (15.5)	11 (15.7)	5 (7.1)	9 (12.9)	25 (35.7)	44.3 ± 16.8
1	134 (29.6)	24 (17.9)	13 (9.7)	15 (11.2)	43 (32.1)	42.8 ± 16.0
2	141 (31.1)	45 (31.9)	15 (10.6)	41 (29.1)	61 (43.3)	38.5 ± 17.3
3	81 (17.9)	31 (38.3)	14 (17.3)	26 (32.1)	50 (61.7)	32.5 ± 15.9
4	27 (6.0)	15 (55.6)	11 (40.7)	12 (44.4)	17 (63.0)	32.7 ± 16.3
3–4	108 (23.8)	46 (42.6)	25 (23.1)	38 (35.2)	67 (62.0)	32.6 ± 16.0

Values are n (%) or mean ± SD. The 3–4 category is included to match the planned figure category, and rows 0, 1, 2, 3, and 4 are mutually exclusive. The primary composite outcome was defined as shoulder-hand syndrome or clinically significant hand edema during inpatient rehabilitation. FMA-UE, Fugl-Meyer Assessment of the Upper Extremity; SD, standard deviation.

Table S7. Pairwise Spearman Correlations among Biomarkers

Biomarker pair	Spearman ρ	p value
Serum zinc vs albumin	0.161	<0.001
Serum zinc vs C-reactive protein	-0.149	0.002
Serum zinc vs neutrophil-to-lymphocyte ratio	-0.221	<0.001
Albumin vs C-reactive protein	-0.074	0.116
Albumin vs neutrophil-to-lymphocyte ratio	-0.137	0.003
C-reactive protein vs neutrophil-to-lymphocyte ratio	0.078	0.096

Spearman rank correlations were calculated using the continuous biomarker values in the analytic cohort (N = 453). The small absolute correlation coefficients indicate limited pairwise overlap among the four components. The reported p values are descriptive and were not adjusted for multiple comparisons.

Table S8. Incremental Model Performance and Bootstrap Internal Validation

Model	Apparent AUROC	Brier score	Nagelkerke R^2	Optimism-corrected AUROC / Brier
Clinical model	0.763	0.164	0.248	0.744 / 0.171
Clinical model + serum zinc	0.778	0.159	0.279	0.758 / 0.167
Clinical model + biomarker	0.776	0.160	0.275	0.756 / 0.168

burden				
Clinical model + serum zinc + biomarker burden	0.781	0.158	0.285	0.758 / 0.167

The clinical model included age, sex, BMI, stroke type, admission FMA-UE score, shoulder subluxation at admission, spasticity at admission, and onset-to-PM&R admission interval. Optimism-corrected AUROC and Brier scores were estimated using 1,000 bootstrap resamples. The clinical-plus-zinc-plus-burden model produced essentially unchanged optimism-corrected AUROC and Brier values compared with the clinical-plus-zinc model. In the model containing both serum zinc and biomarker burden, the adjusted OR for the burden was 1.22 (95% CI, 0.95–1.58; p = 0.125), indicating no clear incremental association beyond zinc after clinical adjustment. AUROC, area under the receiver operating characteristic curve; BMI, body mass index; FMA-UE, Fugl-Meyer assessment of the upper extremity; OR, odds ratio; PM&R, Physical Medicine and Rehabilitation.

Table S9. Temporal Sensitivity Analyses

Analysis	Patients / events	Adjusted OR (95% CI)	p value
Primary analytic cohort	453 / 126	1.43 (1.15–1.77)	0.001
No primary outcome at PM&R admission; biomarker profile preceded any subsequently documented event	425 / 98	1.42 (1.12–1.79)	0.004
Biomarker profile clearly preceded documented outcome in event cases; all non-event patients retained	442 / 115	1.41 (1.13–1.76)	0.003
Biomarker profile obtained on or before PM&R admission	310 / 90	1.43 (1.11–1.85)	0.006

All temporal sensitivity models used the same covariates and HC3 robust standard errors as the primary model. The selected biomarker panel was obtained before PM&R admission in 205 patients (45.3%), on the admission day in 105 (23.2%), and after admission in 143 (31.6%). Twenty-eight patients had the primary composite outcome documented at PM&R admission. Outcome dates refer to the first documented dates in the retrospective clinical record and may not represent exact symptom-onset dates. CI, confidence interval; OR, odds ratio; PM&R, Physical Medicine and Rehabilitation.
